# Ethylate of Soda as a Caustic

**Published:** 1881-12

**Authors:** 


### ETHYLATE OF SODA AS A CAUSTIC.

Dr. R. J. Levis (*Philadelphia Med. Times*, September 24, 1881), claims that ethylate of soda is well adapted to caustic purposes. It is made in the following manner: Half an ounce of rectified alcohol (specific gravity 0.975) is put into a two-ounce test tube, set up in a bath of cold water. To the alcohol, some cuttings of pure sodium are added. Hydrogen at once escapes. Sodium is added till the gas ceases to escape, when the water is warmed to 100° F., and a little more sodium added till the gas ceases to escape; the liquid is then cooled down to 50° F., and half an ounce more alcohol added. This gives a solution of proper strength for a caustic. A glass rod dipped into the solution is recommended as the best mode of application.

